# A Comparative Analysis of International Dog Owner Education Programmes

**DOI:** 10.3390/ani16030370

**Published:** 2026-01-23

**Authors:** Hee Yong Kang, Song Yi Lee

**Affiliations:** Department of Counselling and Coaching, Dongguk University, Seoul 04620, Republic of Korea; khy@dogdays.kr

**Keywords:** dog owner education, human–animal interaction, canine behaviour theory, attachment theory, cognitive–behavioural coaching, comparative case study, guardianship, animal welfare

## Abstract

Dogs increasingly function as relational beings, shaping their guardians’ emotional well-being and daily routines. Consequently, dog owner education has expanded beyond behaviour-focused training toward integrative approaches that address the emotional, relational, and cognitive dimensions of the human–dog relationship. Despite this shift, international comparative research on the organisation and institutionalisation of dog owner education remains limited. The study applies a qualitative exploratory comparative case study to examine systems in the United States, the United Kingdom, Germany, Japan, and Australia, identifying national patterns and shared components to inform context-appropriate frameworks, particularly in South Korea. The study examines legal and policy documents, institutional guidelines, and standardised education programmes that function as national or de facto standards using document and content analysis. It integrates within- and cross-case comparisons and interprets findings through a framework drawing on human–animal interaction, attachment, canine behaviour, and cognitive–behavioural coaching theories. The analysis reveals five shared components of behaviour change: guardian responsibility and animal welfare, science-based positive reinforcement, early socialisation and prevention, a balance between standardisation and individualisation, and guardians’ emotional and relational engagement. These findings suggest that dog owner education functions as an integrated system that supports responsible guardianship and stable human–dog relationships across sociocultural contexts.

## Simple Summary

Dogs are increasingly recognised as relational beings that contribute to their guardians’ emotional well-being and the structure of everyday life. In response, dog owner education has expanded beyond traditional behaviour training to encompass emotional, relational, and cognitive dimensions of the human–dog relationship. However, limited research has examined how dog owner education systems are organised and institutionalised across different countries or whether they share common principles despite variations in cultural and policy contexts. To address this gap, the present study compared dog owner education programmes in five countries—the United States, the United Kingdom, Germany, Japan, and Australia—with a focus on national standards, policies, and institutional guidelines. Although each country has developed its education system within a distinct social and legal framework, several shared elements were identified across all cases. These included a strong emphasis on owner responsibility and animal welfare, science-based training grounded in positive reinforcement, early socialisation and prevention-oriented approaches, a balance between standardised guidance and individual needs, and attention to guardians’ emotional and relational engagement with their dogs. Overall, the findings suggest that dog owner education operates not merely as skills-based training but as an integrated educational system that supports responsible guardianship and stable human–dog relationships. These insights provide a foundation for the development of context-appropriate dog owner education frameworks, particularly in countries where such systems are still in the early stages of development.

## 1. Introduction

### 1.1. Background and Rationale

In contemporary society, people increasingly recognise companion dogs as relational beings that contribute to their guardians’ emotional well-being, social connectedness, and the structuring of daily life [1]. A growing body of research demonstrates that everyday interactions with dogs reduce stress, improve emotional regulation, and strengthen social bonds, indicating that human–dog relationships extend beyond functional roles towards emotionally and relationally grounded partnerships [2,3].

This shift has significant implications for the orientation of dog owner education. Traditional approaches have largely emphasised obedience training and behavioural correction, but critics argue that such skill-centred models fail to address the emotional and relational dimensions of the owner–dog relationship. Recent scholarship, therefore, calls for reconceptualising dog owner education as a comprehensive system that integrates knowledge of canine behaviour with guardians’ emotional sensitivity, relational competence, reflective caregiving attitudes, and ethical responsibility [4].

Despite these developments, institutional practice in South Korea has not yet fully aligned with this expanded framework. Providers mainly deliver education through private training centres, informal programmes, or online information sources, whereas public education systems and legally standardised frameworks remain underdeveloped [5]. Consequently, guardians show wide variation in knowledge, attitudes, and practices, and regulators struggle to ensure consistent educational quality [6].

These structural limitations produce tangible social outcomes. Public complaints about companion dogs in South Korea largely involve aggression, noise, and hygiene issues [7], while national statistics report tens of thousands of abandoned dogs each year [8]. These patterns suggest that insufficient guardian education—particularly in training knowledge and socialisation—drives problem behaviours and increases the risk of relinquishment. Moreover, dog owner education in South Korea largely focuses on technical training skills, with comparatively limited attention to emotional reflection, relational bonding, and the development of guardians’ psychological and behavioural capacities [6,9], which can destabilise owner–dog relationships and undermine long-term responsible guardianship.

Overall, dog owner education in South Korea operates through a predominantly private, skill-oriented, and fragmented system that correlates with abandonment, safety incidents, and community conflict. These conditions underscore the need for legal and institutional frameworks that support an integrated dog owner education system encompassing emotional, behavioural, and relational dimensions. From this perspective, systematic international comparison offers a critical foundation for shaping future development in the Korean context.

### 1.2. Global Trends in Dog Owner Education

Across countries, policymakers and practitioners have increasingly institutionalised dog owner education as a multidimensional system integrating animal welfare, behaviour management, and guardian responsibility, rather than treating it as a series of isolated training interventions. This shift reflects a broader international trend towards conceptualising dog owner education as a comprehensive educational framework that supports responsible guardianship and stable human–dog relationships.

In the United States, representative tiered education models such as the Socialisation, Training, Activity, and Responsible Owner (S.T.A.R.) and Puppy and Canine Good Citizen (CGC) programmes, developed by the American Kennel Club (AKC), emphasise early socialisation, public manners, and responsible guardianship [10,11]. In parallel, professional organisations, including the Association of Professional Dog Trainers (APDT) and the Animal Behaviour College (ABC), offer standardised curricula and certification pathways. Although the law does not mandate participation, these private standards collectively function as a de facto national dog owner education system.

In the United Kingdom, The Kennel Club’s Good Citizen Dog Training Scheme (GCDTS) is a structured, stage-based education framework that progresses from foundational socialisation to advanced public behaviour and guardian responsibility [12]. Complementing this system, the Royal Society for the Prevention of Cruelty to Animals’ (RSPCA) DogKind initiative integrates education with public campaigns that promote welfare-oriented interpretations of canine behaviour and shifts in guardian attitudes towards emotional understanding and responsible caregiving [13,14].

Germany illustrates a hybrid model that combines legal regulation and private certification. Federal welfare regulations, including the Tierschutz-Hundeverordnung (German Animal Welfare Dog Ordinance), establish minimum standards for responsible ownership [15], while regional systems such as the Sachkundenachweis (proof of expertise for dog owners) require education and assessment under specific conditions. Alongside these legal frameworks, private certifications such as those issued by the Verband für das Deutsche Hundewesen (VDH, “German Kennel Club”) assess guardians’ knowledge and public responsibility through theoretical instruction and practical evaluation [16,17].

In Japan, local governments and animal welfare centres primarily implement dog owner education through community-based, daily-life-oriented systems guided by national welfare principles [18]. Public campaigns and regional programmes emphasise responsible guardianship, behaviour management, and prevention. Empirical evidence suggests that these initiatives have increased guardians’ sensitivity to canine emotional signals and behavioural contexts [19].

Researchers often cite Australia as a country with a well-established welfare- and behaviour-based framework for dog owner education. National strategies such as the Australian Animal Welfare Strategy set overarching welfare and responsibility guidelines [20], while institutions such as the Delta Institute deliver training grounded in contemporary behavioural science and positive reinforcement. Empirical findings further indicate that online-based education effectively enhances guardians’ knowledge and attitudes towards behaviour management [21].

Despite differences in institutional design and cultural context, several shared orientations emerge across countries. Dog owner education consistently emphasises ethical responsibility, emotional sensitivity, and the development of stable human–dog relationships, reflecting evidence that links guardians’ emotional states and caregiving attitudes to relationship quality [2,22]. Collectively, these trends indicate an international convergence towards integrated education systems that extend beyond the transmission of technical skills to foster sustainable behavioural change and relational responsibility among guardians.

### 1.3. Theoretical Framework

Previous research on dog owner education in South Korea largely emphasises behaviour correction-oriented approaches, while integrative perspectives addressing emotional interaction within the owner–dog relationship, attachment structures, guardians’ cognitive and emotional regulation capacities, and the scientific principles underlying training practices remain limited [6]. This gap highlights the need for a multidimensional theoretical framework capable of interpreting dog owner education across emotional, behavioural, relational, and cognitive dimensions. Accordingly, the present study adopts human–animal interaction (HAI) theory, attachment theory, cognitive–behavioural coaching (CBC), and canine behaviour theory as complementary analytical lenses.

HAI theory emphasises that emotional and neurophysiological interactions between dogs and their guardians reduce stress, regulate emotions, and provide social connection [2,23]. Empirical findings demonstrating that guardians’ emotional states and interaction styles influence dogs’ behaviour and stress responses (Sundman et al., 2019 [24]) suggest that dog owner education should incorporate emotional sensitivity and interaction regulation alongside behavioural instruction.

Attachment theory conceptualises the guardian–dog relationship as an attachment system grounded in emotional bonding and responsiveness [25]. Research shows that dogs use their guardians as a secure base and exhibit attachment behaviours structurally comparable to those observed in human infants [22,26]. Moreover, studies linking guardians’ caregiving attitudes and attachment styles to dogs’ problem behaviours, social competence, and emotional stability [27,28,29] provide a theoretical basis for interpreting relationship-oriented educational components.

CBC focuses on the interaction of cognition, emotion, and behaviour to promote self-efficacy, emotional regulation, and behavioural change [30,31]. This study does not treat CBC as an operational model formally adopted by national programmes; instead, it uses CBC as an analytical lens to interpret educational elements reflected in programme documents, including goal setting, staged learning processes, feedback mechanisms, self-regulation strategies, and mechanisms facilitating behavioural change.

Canine behaviour theory explains learning and behaviour change through conditioning, reinforcement, and socialisation [32,33]. Contemporary dog owner education increasingly adopts positive reinforcement and consistency as core principles. At the same time, evidence showing that guardians’ interaction styles and emotional responsiveness influence dogs’ behavioural outcomes [27,28] underscores the importance of integrating behavioural theory with emotional and relational dimensions.

Together, these four perspectives provide an integrated framework for analysing dog owner education systems and systematically identifying shared principles and national differences.

### 1.4. Research Objectives and Research Questions

This study compares and analyses dog owner education programmes in the United States, the United Kingdom, Germany, Japan, and Australia to examine how institutions design and operate these programmes within distinct legal, policy, and organisational contexts. Through cross-national comparison, the study identifies core educational components and underlying structural and psychosocial mechanisms embedded in national programmes. The analysis interprets these findings through HAI theory, attachment theory, canine behaviour theory, and cognitive–behavioural coaching, with the ultimate goal of establishing empirical and theoretical foundations for designing dog owner education systems appropriate to the sociocultural context of South Korea.

Accordingly, we formulated the following research questions (RQs):

1. What institutional characteristics define dog owner education programmes in each country?
2. What core educational components commonly appear across national dog owner education programmes?
3. How do the human–animal interaction theory, attachment theory, cognitive–behavioural coaching, and canine behaviour theory interpret the identified core components?

## 2. Methods

### 2.1. Study Design

This study employs a qualitative, exploratory, comparative case study design. Case study methodology effectively supports the examination of complex institutional and cultural phenomena within real-life contexts [34]. In particular, a multiple-case design at the national level enables systematic identification of shared patterns and contextual differences across diverse institutional arrangements [35].

The study adopts an international comparative approach because the degree of institutionalisation of dog owner education varies substantially across countries. In South Korea, dog owner education largely depends on private sector initiatives, whereas public-level standardisation and legal frameworks remain underdeveloped [6]. Accordingly, this study analyses international cases to establish comparative reference points and theoretical implications for developing a context-appropriate dog owner education system in South Korea.

The study design incorporates three key methodological features.

First, the study uses an exploratory approach that prioritises identifying recurring structures and patterns within institutional documents and standardised education programmes rather than testing predefined hypotheses. This approach enables the identification of common components and distinctive national characteristics [36].

Second, the study employs an interpretive theorising strategy. After identifying themes and patterns through document analysis and cross-case comparison, we applied the HAI theory, attachment theory, canine behaviour theory, and CBC as interpretive lenses. In particular, we used CBC selectively to interpret elements such as goal setting, staged implementation, feedback mechanisms, and self-regulation processes. Importantly, we did not treat CBC as an officially adopted operational model by national programmes; instead, we maintained a clear distinction between documented programme content and analytical interpretation.

Third, we conducted an interpretive depth analysis to move beyond surface-level comparison. This approach facilitated a contextual examination of how national education systems integrate emotional intervention, behavioural change processes, and relationship-centred principles within their institutional frameworks [36,37].

Accordingly, we treated the five countries as national-level cases. We systematically compared institutional structures and educational components and subsequently interpreted shared core elements identified across cases in relation to the study’s theoretical framework.

### 2.2. Research Targets and Data Collection

#### 2.2.1. Criteria for Case Selection

This study’s unit of analysis is the institutionalised structure of dog owner education rather than the effectiveness of individual programmes. We employed a purposive sampling strategy, guided by three criteria and following the principles outlined by Stake [35] and Yin [34].

First, selected programmes or systems had to demonstrate institutional establishment through national legislation, public policy, or credible private organisations and function as official or de facto national standards. This criterion included legally mandated systems and widely recognised private programmes that operate as quasi-national benchmarks.

Second, dog owner education content had to extend beyond technical training skills and structurally incorporate psychosocial dimensions, including guardian responsibility, animal welfare and ethics, emotional interaction, and social coexistence. This criterion reflects the study’s conceptualisation of dog owner education as a multidimensional educational system rather than a behaviour training intervention alone.

Third, the study required access to primary documentary sources, such as legal texts, policy documents, institutional guidelines, and programme manuals. We did not treat academic studies as primary analytical units for institutional comparison but used them as secondary interpretive sources to provide contextual background and support interpretation. These criteria align with Bowen’s [38] principles of document analysis, emphasising triangulation, contextual interpretation, and analytical transparency.

Based on these criteria, we selected five countries as comparative cases: the United States, the United Kingdom, Germany, Japan, and Australia.

#### 2.2.2. Comparative Cases and Data Composition

The primary analytical materials consisted of documents and guidelines that function as official or de facto national standards for dog owner education in each country, while academic studies served as supplementary interpretive sources. This approach reduced the imbalance arising from cross-national differences in academic output and enhanced comparability by focusing on institutionalised education systems themselves. The study ensured data credibility through source triangulation [38].

In the United States, where no federally mandated system exists, we examined the American Kennel Club’s S.T.A.R. and Puppy and Canine Good Citizen programmes as de facto national standards, supplemented by professional guidelines issued by the Association of Professional Dog Trainers (APDT) [39] and Animal Behaviour College (ABC) [40]. We used academic research to contextualise implementation practices (e.g., Johnson and Wynne [41]).

In the United Kingdom, we centred the analysis on the Animal Welfare Act 2006 and the Five Welfare Needs framework, with The Kennel Club’s Good Citizen Dog Training Scheme (GCDTS) [12] and the RSPCA’s [42] DogKind programme serving as core materials. Studies by Philpotts et al. [13,14,43] supported the interpretation of the links between education, welfare standards, and guardian responsibility.

In Germany, we examined federal regulations such as the Tierschutz-Hundeverordnung [15], state-level Sachkundenachweis requirements, and the VDH Hundeführerschein (dog owner competency certificate) to reflect a system that combines legal regulation with private certification. Academic studies supported the interpretation of the social and behavioural context [16,17].

In Japan, where national mandates are limited, analysis focused on guidelines issued by the Ministry of the Environment [18], local government materials (e.g., Tokyo), and educational resources from the Japan Animal Welfare Society (JAWS) [44]. We used the study by Yamada et al. [19] to supplement the interpretation of guardian behaviour and perceptions.

In Australia, analysis centred on national welfare strategies associated with the Australian Animal Welfare Strategy (AAWS) [20], alongside vocational education programmes provided by the Delta Institute [45,46,47] and materials from RSPCA Australia [48], reflecting an integrated public–professional education model. We referred to Napier et al. [21] to interpret education delivery modes.

By cross-verifying legal, policy, institutional, and academic sources, we established a structured documentary corpus. The final dataset comprised eight legal and regulatory documents, eight policy and institutional materials, and eight academic studies. Table 1 presents country-specific data composition and selection rationales.

#### 2.2.3. Data Collection Procedures

Data collection lasted approximately eight months, from April to November 2025. To ensure methodological rigour in this document-based qualitative comparative case study, we applied the principles of multiple data sources and transparent data management [34,38]. Data collection and analysis proceeded iteratively to enhance credibility and analytical consistency.

The data collection process comprised four stages.

Stage 1: Preliminary exploration and screening.

We conducted an initial search using academic databases, including Scopus, the Research Information Sharing Service (RISS), and Google Scholar, as well as official websites of public authorities and professional organisations in each country. Search terms included combinations of “dog owner education,” “responsible ownership,” “animal welfare programme,” “training scheme,” and “canine behaviour education,” paired with country names. The initial search resulted in approximately 120 documents. We excluded commercial materials, personal blogs, sources with unclear authorship, and documents with limited relevance. We also excluded documents published prior to 2015 but included legal frameworks and national policy documents with enduring structural influence.

Stage 2: Collection of official documents and institutional guidelines.

We collected legal and institutional documents functioning as official or de facto national standards for dog owner education and treated them as primary analytical materials. We classified documents as national standards when they met three criteria: (1) governmental authorities or widely adopted professional organisations issued them; (2) they explicitly specified educational objectives, content, and evaluation criteria; and (3) they functioned as reference frameworks at the national level. Based on these criteria, we collected recent policy documents, guidelines, and programme manuals from organisations in the United States (AKC, APDT), the United Kingdom (The Kennel Club, RSPCA), Germany (BMEL, VDH), Japan (Ministry of the Environment, Tokyo Animal Welfare Centre, JAWS), and Australia (Australian Government, RSPCA Australia, Delta Institute). In federal systems, we selected representative federal- and state-level systems frequently cited in policy and research literature.

Stage 3: Integration of academic studies and preliminary coding.

We collected relevant academic studies as supplementary interpretive sources rather than primary objects of institutional comparison. We used these studies to contextualise system design and educational effects. Key sources included Johnson and Wynne [41], Philpotts et al. [13,14,43], Küeper and Merle [16], Rauch et al. [17], Yamada et al. [19], and Napier et al. [21]. Preliminary coding followed the content analysis procedures of Elo and Kyngäs [49], identifying recurrent meaning units related to institutional structure, educational objectives, delivery mechanisms, psychological and behavioural components, evaluation systems, and expressions of responsibility and ethics.

Stage 4: Cross-verification and interpretive review.

We applied triangulation to examine consistency across legal and policy documents, institutional guidelines, and academic studies [38]. We systematically reviewed differences in terminology, publication timing, and country-specific conceptual usage. External reviewers translated and back-translated documents originally written in German and Japanese to minimise semantic distortion.

In the final phase, we catalogued country-specific document sets by document type, issuing body, year of publication, and major thematic domains. Appendix A and Appendix B present the resulting national document corpora and summaries of the screening process, which served as the empirical basis for the within- and cross-case analyses reported in Section 3.

#### 2.2.4. Data Analysis Procedures

Data analysis proceeded through an integrated sequence of document analysis, content analysis, within-case analysis, cross-case comparison, and theory-based interpretation [34,35]. The analytical process consisted of five stages.

Stage 1: Data familiarisation and preliminary coding.

We repeatedly reviewed all documents to ensure contextual familiarity. We inductively generated preliminary codes by identifying recurrent meaning units related to educational objectives, programme stages, delivery systems, evaluation and certification mechanisms, behavioural norms, welfare and ethical orientations, and psychological or behavioural expressions. The research questions (RQ1–RQ3) guided analytical focus, while open coding followed data-driven principles [49]. To minimise source-specific bias, we coded across multiple document types, including legal and policy documents, institutional guidelines, manuals, and academic literature.

Stage 2: Category development and analytical framework refinement.

We integrated and refined preliminary codes based on semantic similarity, functional roles, and structural positioning within education systems. We clarified hierarchical relationships among codes through comparative matrix techniques [50]. Through this process, we established seven overarching categories for cross-national comparison: (1) institutional structure, (2) educational objectives and stages, (3) delivery and implementation agents, (4) evaluation and certification systems, (5) psychological foundations, (6) standardisation–individualisation balance, and (7) public orientation, ethics, and responsibility. Appendix C presents the finalised codebook.

Stage 3: Within-case analysis.

We treated each country as an independent case and developed in-depth descriptions focusing on institutional contexts, modes of institutionalisation, programme structures, implementation systems, evaluation mechanisms, and the integration of psychological and behavioural elements [35]. This stage directly addresses RQ1 and establishes the analytical basis for cross-case comparison.

Stage 4: Cross-case comparison.

We synthesised the findings from the within-case analyses to identify shared core components of dog owner education across countries. We derived core components (RQ2) empirically rather than assuming a priori and classified components as “shared” only when they met all three criteria:

1. They appeared in at least four of the five countries, either explicitly or through conceptually equivalent expressions;
2. At least two different source types (e.g., legal documents, institutional guidelines, policy materials, or academic studies) confirmed the presence of the component;
3. They substantively informed educational objectives, structural design, delivery methods, or evaluation mechanisms rather than appearing solely as declarative values.

We employed pattern matching and explanatory comparison procedures and organised results into comparative matrices to ensure traceability to source documents [34,35].

Stage 5: Theory-based integrative interpretation.

In the final stage, we interpreted the shared core components using human–animal interaction (HAI) theory, attachment theory, cognitive–behavioural coaching (CBC), and canine behaviour theory as analytical lenses. We applied these frameworks as interpretive tools to clarify the functions and meanings of the identified components and elucidate the psychological, behavioural, and relational mechanisms underlying dog owner education systems, thereby addressing RQ3 with interpretive coherence.

## 3. Results

This section reports the study’s findings in line with the research questions. We present the results through within-case analyses of national dog owner education systems (RQ1), followed by a cross-case comparison to identify shared core components (RQ2) and a theory-based interpretation of these components (RQ3).

### 3.1. Within-Case Analysis: Institutional Characteristics (RQ1)

Dog owner education systems demonstrated substantial variation across countries in terms of legal grounding, implementing bodies, delivery mechanisms, and the structuring of educational content. To address RQ1—“What institutional characteristics define dog owner education programmes in each country?”—we analysed the United States, the United Kingdom, Germany, Japan, and Australia as independent national-level cases.

Table 2 summarises the institutional structures and operational characteristics of dog owner education across the five countries using the shared analytical categories established in the Section 2. The table provides a concise overview of cross-national similarities and differences and serves as a structural reference for the detailed within-case descriptions and the subsequent cross-case comparison addressing RQ2.

#### 3.1.1. Institutional Structure

Dog owner education programmes across the five countries exhibit distinct institutional structures shaped by national legal frameworks, the distribution of responsibilities between public and private actors, and the organisation of education and certification systems.

In the United States, dog owner education operates within a privately led, self-regulatory model, as there are no federally mandated legal requirements. Organisations such as the American Kennel Club, the Association of Professional Dog Trainers, and Animal Behaviour College function as de facto standard-setting bodies, guiding education through voluntary participation and social norms rather than statutory regulation [10,11,39,51]. Practitioners widely recognise the AKC’s Canine Good Citizen (CGC) programme [10] as a quasi-standard for assessing guardian competence and responsible ownership.

In the United Kingdom, a legal framework grounds dog owner education. The Animal Welfare Act 2006 establishes guardians’ legal duty of care, while the Five Welfare Needs framework defines minimum welfare standards [52]. Within this context, The Kennel Club’s Good Citizen Dog Training Scheme disseminates welfare-oriented and responsibility-focused education aligned with public etiquette norms, representing a public–private linkage model that integrates statutory obligations and private standards [13,42].

Germany demonstrates the highest level of legal institutionalisation among the cases examined. Federal legislation, including the Tierschutzgesetz (German Animal Welfare Act) and the Tierschutz-Hundeverordnung, defines principles of dog guardianship and education [15]. At the state level, regulations such as Section 3 of the Lower Saxony Dog Act require certain first-time dog owners to obtain a Sachkundenachweis through theoretical and practical assessments, typically linked to the early phase of ownership, although implementation details vary by state [51]. This structure reflects a regulation-centred model in which statutory requirements operate alongside private certification systems (e.g., VDH).

In Japan, national animal welfare and management guidelines issued by the Ministry of the Environment guide dog owner education, whereas local governments largely undertake programme design and delivery. Although there is limited legally mandated education, municipal programmes emphasise community coexistence, prevention of problem behaviours, and socialisation. Overall, Japan’s model is decentralised and community-oriented, prioritising practical adaptation to local living environments [19].

Australia exhibits an integrated public–professional model that combines animal welfare policy frameworks with vocational education systems. Although Australia has formally concluded its Australian Animal Welfare Strategy (AAWS) [20], state and territory governments continue to implement region-specific welfare legislation and educational guidelines. RSPCA Australia provides influential national education materials that link policy and practice, whereas institutions such as the Delta Institute deliver training and certification within the national vocational education and training (VET) framework, supporting professional standards in dog owner education [45,46,47,48].

In summary, the institutional structures of dog owner education vary across countries: the United States and the United Kingdom rely primarily on private standards or public–private linkage models; Germany employs a regulation-centred approach; Japan adopts a decentralised, local-government-led system; and Australia integrates welfare policy, vocational education, and public institutions in a unified framework.

#### 3.1.2. Educational Goals and Programme Structure

Across the five countries examined, dog owner education programmes share several core educational goals, including the prevention of problem behaviours, the promotion of safe and stable companion dog living, the development of socialisation and basic manners, and the reinforcement of guardian responsibility. Differences arise, however, in how countries prioritise and structure these goals into educational stages, reflecting each country’s institutional context and policy orientation.

In the United States, dog owner education primarily emphasises responsible ownership and basic behavioural stability through staged training pathways. Programmes such as the American Kennel Club’s S.T.A.R. Puppy, Canine Good Citizen (CGC), and Community Canine progressively reinforce socialisation, environmental adaptation, public manners, and guardianship management practices [10,11]. Institutions frame educational outcomes in terms of canine behaviour and in guardians’ responsible conduct in everyday and public contexts.

In the United Kingdom, educational goals extend beyond behavioural training to include awareness of welfare, emotional bonding, and social responsibility. The Good Citizen Dog Training Scheme (GCDTS) adopts a four-stage structure (Puppy Foundation to Gold), in which they assess canine learning outcomes and guardians’ caregiving practices [13]. Complementarily, the RSPCA’s DogKind programme emphasises relationship-centred education by foregrounding guardians’ emotional regulation and interpretation of dogs’ emotional and behavioural signals [48].

In Germany, dog owner education objectives closely align with legal obligations. The Sachkundenachweis system under the Lower Saxony Dog Act [51] requires eligible guardians to demonstrate competence through theoretical and practical assessments covering canine behaviour, management, legal responsibilities, and public control. Although not organised as a multi-tiered programme, this structure follows a sequential process of instruction, assessment, and application, with institutional priorities centred on behaviour prevention, aggression reduction, and public safety.

In Japan, education is largely at the municipal level, resulting in programmes that prioritise adaptation to daily life, the prevention of problem behaviour, socialisation, and community coexistence. Education typically consists of short, repetitive modules such as puppy socialisation classes, basic manners training, and behavioural counselling, with some programmes also addressing guardian–dog emotional attunement and guardian stress awareness [19].

In Australia, dog owner education emphasises science-based training, welfare-focused care, socialisation, and early prevention of behavioural issues. RSPCA Australia structures its educational materials around guardian competencies such as environmental management, stress-signal recognition, and welfare-compliant interaction [42]. Professional education providers, including the Delta Institute, offer staged modules grounded in learning theory and positive reinforcement, partially linking general owner education with professional training pathways [45,46,47].

Overall, the United States adopts a behaviour-centred, staged training model; the United Kingdom integrates behavioural, emotional, and welfare goals within a multi-level framework; Germany emphasises legally grounded knowledge and skill acquisition; Japan operates a decentralised, practice-oriented modular education; and Australia presents an integrated, science-based, welfare-oriented structure. These variations reflect each country’s underlying animal welfare philosophy and policy priorities as institutionalised through dog owner education.

#### 3.1.3. Delivery Systems and Implementing Bodies

Dog owner education systems across the five countries differ substantially in their delivery structures and configurations of implementing bodies, reflecting national institutional arrangements that shape public involvement, professional regulation, and accessibility.

In the United States, private sector organisations play a dominant role in delivering dog owner education. AKC-certified trainers, APDT-affiliated professionals, and institutions such as the Animal Behaviour College serve as primary providers [10,11,39]. While public authorities play a limited role, professional organisations contribute to quality assurance by setting ethical and educational standards within a largely market-oriented system.

The United Kingdom operates a more clearly institutionalised public–private partnership model. National organisations such as The Kennel Club and the RSPCA provide accreditation, guidelines, and public welfare campaigns, while certified educators deliver programmes locally [48]. This structure combines decentralised implementation with centralised coordination, supporting consistency in educational delivery.

Germany’s delivery system has strong public oversight. Federal state (Länder) authorities supervise implementation, while certified trainers deliver education. Some states, including Lower Saxony, institutionally separate instructional provision and assessment for the Sachkundenachweis, reinforcing regulatory independence between education and evaluation [51].

Japan employs a municipality-centred model in which local animal welfare centres, public officials, veterinarians, and private trainers collaborate to deliver education. Although the Ministry of the Environment issues national guidelines, local communities implement them through individualised consultation and preventive support, resulting in flexibility and inter-municipal variation [19].

Australia demonstrates an integrated public–professional delivery system. RSPCA Australia provides national education resources and direct programmes, while vocational institutions such as the Delta Institute and TAFE train accredited professionals who subsequently implement owner education in practice [42,45,46,47]. In several jurisdictions, trainer registration or approval schemes support quality control.

In summary, we can categorise delivery systems as follows: a private professional-led model in the United States, a public–private collaborative model in the United Kingdom, a public oversight-centred regulatory model in Germany, a municipality-based collaborative model in Japan, and an integrated public–professional model in Australia. These patterns illustrate how national governance structures shape the practical implementation of dog owner education.

#### 3.1.4. Assessment and Certification Systems

Dog owner education programmes across the five countries differ substantially in the degree of institutionalisation of assessment and certification, reflecting variations in credibility, enforceability, and mechanisms for quality assurance.

Although the United States does not legally mandate dog owner education, the American Kennel Club’s Canine Good Citizen (CGC) test functions as a widely recognised de facto national benchmark. The CGC evaluates guardians’ management practices and dogs’ behavioural stability in public contexts and has acquired strong social legitimacy despite its voluntary nature [10,11]. In parallel, professional organisations such as the Association of Professional Dog Trainers (APDT) contribute to quality assurance by maintaining ethical and competency standards for educators [39].

Assessment systems in the United Kingdom more explicitly align with public welfare principles. The Kennel Club’s Good Citizen Dog Training Scheme (GCDTS) employs clearly structured achievement levels, with standardised criteria assessing welfare compliance, socialisation, and public behaviour across progressive stages [13]. Complementarily, the RSPCA’s DogKind programme incorporates guardians’ emotional responsiveness and welfare-oriented attitudes as evaluative dimensions, extending assessment beyond technical performance to relational and attitudinal change [48].

Germany represents the most legally institutionalised assessment model. Under the Lower Saxony Dog Act [51], specified categories of guardians—particularly first-time dog owners—must obtain a Sachkundenachweis, comprising theoretical and practical examinations. Authorised institutions administer the assessments and emphasise public safety, behavioural stability, and compliance with statutory animal welfare principles [15].

Japan has limited national-level standardised certification; instead, municipalities primarily conduct assessments. Local programmes rely on participation records, counselling-based evaluations, and qualitative monitoring of behavioural improvement and home management practices [19]. Rather than formal certification, the emphasis lies on practical outcomes and community coexistence.

Australia employs a multi-layered system integrating national vocational qualifications and institutional assessment. Certificate IV in Animal Behaviour and Training, delivered through the VET system, provides nationally recognised professional certification for educators [45,46,47]. Within guardian education, organisations such as RSPCA Australia conduct competency-based assessments and informal evaluations, focusing on welfare compliance, socialisation, and problem behaviour prevention [42].

Overall, we can summarise assessment and certification systems as follows: the United States and the United Kingdom rely on socially trusted, privately led certification frameworks; Germany adopts a conditionally mandatory, legally enforced model; Japan emphasises community-based, qualitative evaluation; and Australia integrates professional qualification systems with competency-based assessment. Across all cases, assessment practices increasingly extend beyond behavioural skill verification to encompass guardians’ responsibility, welfare awareness, and relational competence.

#### 3.1.5. Psychological Foundations

Despite differences in institutional structures and delivery systems, dog owner education programmes across the five countries consistently incorporate psychological foundations in behavioural science, emotional and relational interactions, and changes in guardians’ cognition and attitudes.

The United States primarily grounds dog owner education in operant conditioning and positive reinforcement. Guidelines issued by the American Kennel Club and the Association of Professional Dog Trainers explicitly discourage punitive or coercive techniques and promote reward-based learning [10,11,39]. Within this framework, guardians’ emotional regulation, understanding of canine emotional states, and relationship quality function as key mediators of behavioural change, with empirical evidence indicating that these relational factors enhance training effectiveness [41].

In the United Kingdom, psychological foundations extend beyond technical training to emphasise emotional communication, stress reduction, and welfare-oriented interaction. The Good Citizen Dog Training Scheme and the RSPCA’s DogKind programme adopt fear-free and welfare-first principles, framing behavioural change as a process facilitated by emotional safety, stress management, and guardians’ attitudinal change [13,48].

Germany has traditionally emphasised behavioural control and public safety in line with statutory animal welfare regulations [15]. However, recent research suggests a gradual shift towards recognising relational and emotional factors, such as guardians’ consistency, predictability, and provision of emotional security, as contributors to behavioural stability [16,17].

In Japan, municipally led programmes adopt pragmatic, life-oriented psychological approaches rather than explicitly articulated theoretical frameworks. Educational content focuses on everyday problem prevention, interpretation of emotional signals, stress reduction, and socialisation, thereby implicitly fostering guardians’ emotional sensitivity and daily management capacities [19].

Australia integrates behavioural science with emotional and relational perspectives. RSPCA Australia emphasises understanding stress signals and applying environmental management strategies to support welfare and behavioural stability, while professional education providers such as the Delta Institute explicitly link learning theory with guardians’ instructional competencies [42,45,46,47].

Overall, across national contexts, dog owner education conceptualises canine behaviour as the outcome of technical training and a dynamic psychological process shaped by emotional states, relationship quality, and guardians’ perceptions and caregiving practices. Across countries, programmes consistently identify positive reinforcement, emotional attunement, stress reduction, and attitudinal change as shared psychological foundations underpinning dog owner education.

#### 3.1.6. Standardisation–Customisation Balance

Across the five countries, dog owner education programmes exhibit different configurations in balancing standardised educational frameworks with customisation tailored to individual guardians and their dogs. These configurations reflect national animal welfare philosophies, public safety priorities, and educational orientations.

In the United States, nationally recognised standardised modules such as the AKC’s S.T.A.R. Puppy and Canine Good Citizen (CGC) programmes provide a common educational framework [10,11]. However, the predominantly private-led training environment allows for substantial customisation in practice, with training approaches adjusted according to individual behavioural concerns, guardians’ caregiving styles, and living environments. The APDT’s ethical guidelines reinforce this flexibility, explicitly emphasising the need to adapt training to the specific guardian–dog relationship [39].

In the United Kingdom, the Good Citizen Dog Training Scheme operates through clearly defined stage-based standards, whereas welfare-oriented initiatives such as the RSPCA’s DogKind incorporate flexibility based on guardians’ emotional states, dogs’ stress levels, and contextual factors [13,48]. As a result, standardised national certification coexists with emotionally and welfare-informed individualisation.

Germany demonstrates the strongest emphasis on regulatory standardisation. Legal requirements associated with the Sachkundenachweis and the management standards stipulated in the Tierschutz-Hundeverordnung function as powerful standardising mechanisms [15,51]. Although institutions permit some adjustment based on canine temperament or risk level, they constrain customisation by prioritising compliance with legally defined criteria.

In Japan, the dominance of municipally operated, community-based programmes results in relatively low nationwide standardisation. At the same time, this decentralised structure enables high levels of customisation aligned with guardians’ lifestyles and dogs’ living environments [19]. Rather than uniform programme structures, Japanese dog owner education prioritises pragmatic, case-based interventions embedded in daily life contexts.

Australia exhibits a hybrid model in which standardised guidelines coexist with contextualised implementation. RSPCA Australia provides nationally consistent welfare and behaviour guidance [42], while regional providers adapt educational delivery to local conditions and individual needs. Similarly, the Delta Institute offers standardised curricula within the national vocational education framework, complemented by individualised feedback and practice-based assessment [45,46].

Overall, the United States, the United Kingdom, and Australia emphasise flexible customisation within standardised frameworks; Germany prioritises strong legal standardisation with limited flexibility; and Japan combines low national standardisation with high levels of life-contextual customisation. These patterns indicate that each country’s institutional context and educational philosophy closely shape the balance between standardisation and customisation in dog owner education.

#### 3.1.7. Ethical Responsibility and Welfare Orientation

Across the five countries examined, dog owner education programmes employ diverse institutional and educational mechanisms to reinforce public responsibility, ethical conduct, and guardians’ accountability. These differences reflect each country’s animal welfare paradigm and societal expectations regarding responsible dog ownership.

In the United States, legal enforceability remains limited; however, private professional organisations play a central role in articulating ethical standards. The American Kennel Club and the Association of Professional Dog Trainers explicitly promote welfare-friendly, non-coercive training principles through professional guidelines and codes of ethics [10,11,39]. The CGC programme further reinforces guardians’ responsibility by incorporating public etiquette, safety management, and responsible ownership behaviours into its certification criteria. Although participation is voluntary, CGC functions as a socially recognised mechanism for strengthening ethical responsibility grounded in shared norms rather than statutory obligation.

The United Kingdom represents the most explicitly institutionalised case in integrating ethical responsibility into dog owner education. The Animal Welfare Act 2006 legally defines guardians’ duty of care, and the Five Welfare Needs framework [52] provides a framework for these principles, while the Good Citizen Dog Training Scheme [13] structurally embeds these principles in its educational goals and assessment criteria. In parallel, the RSPCA’s DogKind programme emphasises emotional safety, recognition of stress signals, and positive interaction, thereby framing guardians’ emotional regulation and relational ethics as core components of responsible ownership [48].

Germany demonstrates the strongest legal institutionalisation of ethical and public responsibility. The Tierschutzgesetz and Tierschutz-Hundeverordnung establish minimum standards for dog care and management while explicitly linking guardians’ negligence or inadequate risk management to public safety concerns [15]. The Lower Saxony Dog Act (NHundG) further mandates guardian certification through the Sachkundenachweis, institutionalising the assessment of guardians’ legal responsibility awareness alongside knowledge and behavioural management competence [52].

In Japan, although formal legal regulation is comparatively limited, municipally led dog owner education programmes consistently emphasise public responsibility through goals such as community coexistence, the prevention of problem behaviours, and social safety [19]. Educational practices prioritise socialisation, basic management principles, and adherence to public norms, with some programmes incorporating relational elements such as understanding guardians’ emotional responses and interpreting canine signals.

Australia’s dog owner education strongly emphasises animal welfare principles and responsible guardianship. RSPCA Australia [42] defines guardians’ responsibility to meet dogs’ environmental, health, behavioural, and emotional welfare needs, positioning problem behaviour prevention as a core guardian obligation. Professional education providers similarly integrate ethical standards and welfare guidelines into training curricula, prioritising welfare protection in behaviour intervention and training practices [45,46,47].

Overall, despite differences in institutional enforcement, dog owner education programmes across all five countries share a common recognition that guardians’ behaviours affect canine welfare, public safety, and community relations. The integration of ethical responsibility, welfare orientation, and public accountability thus constitutes a fundamental and shared dimension of contemporary dog owner education systems.

### 3.2. Cross-Case Comparison: Shared Core Components Across Countries (RQ2)

This section addresses RQ2: “What core components commonly appear across national dog owner education programmes?” Building on the within-case analyses, we conducted a cross-case comparison of dog owner education systems in the United States, the United Kingdom, Germany, Japan, and Australia, using the seven analytical categories defined in the Section 2.

Despite substantial differences in legal frameworks, institutional arrangements, and degrees of regulatory enforcement, the comparative analysis revealed a convergence towards a set of shared orientations and structural principles. Five core components consistently emerged across all five countries: (1) an emphasis on owner responsibility and animal welfare standards, (2) the application of science-based training grounded in positive reinforcement, (3) early socialisation and prevention-oriented interventions, (4) a hybrid structure combining standardised guidance with contextual customisation, and (5) attention to emotional and relational interaction alongside changes in owner attitudes and behaviours.

Importantly, we did not interpret cross-national differences as contradictions of these shared components; instead, we viewed them as variations in how institutions operationalise these components across distinct legal, institutional, and sociocultural contexts. As summarised in Table 3, each country implements these components through different combinations of policy instruments, educational programmes, and institutional actors.

Taken together, these findings indicate that contemporary dog owner education systems across national contexts share common educational and psychosocial principles. This shared foundation provides an empirical basis for the theory-based interpretation of underlying mechanisms presented in RQ3.

#### 3.2.1. Guardian Responsibility and Welfare Standards

Guardian responsibility and compliance with animal welfare standards emerged as a foundational component of dog owner education across all five countries.

In the United Kingdom, the Animal Welfare Act 2006 and the Five Welfare Needs framework legally define guardians’ duty of care. Programmes such as the Good Citizen Dog Training Scheme and RSPCA initiatives systematically embed these principles. Germany similarly institutionalises guardian responsibility and public safety obligations through statutory frameworks such as the Tierschutzgesetz and the NHundG.

In contrast, the United States, Australia, and Japan exhibit lower levels of legal compulsion. Nevertheless, non-governmental standards and educational frameworks developed by organisations such as the AKC, APDT, RSPCA Australia, and local governments consistently emphasise responsibility and welfare principles. All cases frame guardian behaviour as influencing dogs’ physical and emotional welfare, public safety, and community coexistence. While the United Kingdom and Germany primarily rely on legal mandates, the United States, Australia, and Japan promote guardian responsibility through social norms, voluntary participation, and education-based engagement.

#### 3.2.2. Science-Based Training Grounded in Positive Reinforcement

The five countries consistently ground dog owner education in behavioural science, with a shared emphasis on positive reinforcement and non-coercive training approaches. Educational guidelines from the AKC and APDT (United States), GCDTS and DogKind (United Kingdom), RSPCA Australia and the Delta Institute, as well as materials used in Germany and Japan, reflect core principles of operant conditioning and reinforcement-based learning while explicitly discouraging punitive techniques. Despite national differences in programme structure, common patterns include interpreting problem behaviours as outcomes of interactions among the environment, emotion, and learning processes; prioritising reward-based learning; and reducing fear and stress through predictable, consistent guardian responses. These shared features indicate a broad international convergence towards science-based, welfare-oriented training paradigms.

#### 3.2.3. Early Socialisation and Prevention-Oriented Interventions

Early socialisation and prevention-oriented intervention constitute a core educational strategy across all national cases. Programmes such as S.T.A.R. Puppy (United States), the Puppy Foundation stage of the GCDTS (United Kingdom), municipal socialisation initiatives in Japan, socialisation-focused guidelines in Australia, and preparatory education linked to qualification systems in Germany consistently emphasise early exposure, environmental adaptation, and developing basic manners. Rather than prioritising corrective interventions after problem behaviours emerge, dog owner education systems emphasise preventive approaches during early life stages and the initial adoption period. Gradual exposure to novel environments, positive social interactions, consistent guardian responses, and stable attachment relationships are key mechanisms for reducing future behavioural problems and stress.

#### 3.2.4. Parallel Structures of Standardisation and Customisation

All five countries demonstrate a parallel structure in which standardised educational frameworks coexist with context-sensitive customisation. In the United States and the United Kingdom, nationally recognised staged programmes provide standardised benchmarks, while practical implementation allows adaptation based on individual dogs’ characteristics, emotional states, and living environments. Australia similarly combines national welfare guidelines with regionally and institutionally adjusted delivery. Germany places greater emphasis on legal standardisation, resulting in more limited flexibility, whereas Japan exhibits relatively low national standardisation alongside a high degree of municipal- and case-based adaptation. Overall, the coexistence of standardised core criteria and flexible application emerges as a shared structural feature of contemporary dog owner education systems.

#### 3.2.5. Emotion and Relationship-Centred Interaction and Guardian Change

All countries emphasise emotion- and relationship-centred interactions between guardians and dogs, along with changes in guardians’ perceptions and attitudes, as key psychosocial mechanisms in dog owner education. The countries consistently frame education as extending beyond technical skill acquisition to include understanding the emotional and relational meanings underlying canine behaviour, enhancing guardians’ emotional regulation, and fostering mutual trust. Programmes such as DogKind in the United Kingdom and RSPCA Australia explicitly highlight communication signal recognition, stress reduction, and awareness of emotional states. Educational initiatives in the United States and Australia place particular emphasis on changing guardian attitudes, while Japanese programmes focus on emotional attunement through everyday interaction management. Recent educational discourse in Germany similarly reflects growing recognition of emotional and relational stability as contributors to behavioural improvement.

### 3.3. Theory-Based Integrative Interpretation (RQ3)

This section addresses RQ3—“How do the human–animal interaction theory, attachment theory, cognitive–behavioural coaching, and canine behavioural theory interpret the identified core components?”—by providing an integrated theoretical interpretation of the five shared core components identified through cross-case comparison (RQ2). We conceptualise dog owner education as the transmission of training techniques and a process involving changes in owners’ cognition, emotion, and behaviour.

This study distinguishes between theories explicitly reflected in documentary evidence (e.g., legislation, institutional guidelines, and programme manuals) and those applied interpretively to explain the underlying mechanisms of educational functioning. Behavioural theory and HAI theory constitute the primary theoretical foundations explicitly documented across national contexts, whereas we apply CBC as an interpretive analytical lens to explain owner change processes embedded within educational structures. Attachment theory and HAI theory further serve as complementary frameworks where emotional and relational processes are salient. Table 4 summarises this structure of theoretical application.

#### 3.3.1. Guardian Responsibility and Welfare Standards

HAI theory provides a direct foundation for owner responsibility and welfare standards by demonstrating how owners’ interaction styles influence dogs’ emotional states, behavioural stability, and welfare [2,23]. Documents such as the UK Animal Welfare Act 2006, Germany’s Sachkundenachweis, and the AKC’s Canine Good Citizen criteria explicitly institutionalise this perspective. We apply CBC interpretively to explain how documented elements—such as goal setting, responsibility awareness, and evaluation criteria—functionally correspond to CBC principles of goal clarification, self-efficacy enhancement, and action-oriented behaviour change [53,54].

#### 3.3.2. Science-Based Training Grounded in Positive Reinforcement

Positive reinforcement-based training represents the component with the clearest direct grounding in behavioural theory. Programmes across all five countries explicitly articulate principles of operant conditioning and reinforcement [32,55], alongside the rejection of punitive methods. We apply CBC at an interpretive level, as its cycle of behavioural experimentation, feedback, and adjustment closely parallels reinforcement-based learning processes [30,31]. However, because owner education documents do not explicitly cite CBC, we interpret positive reinforcement training as behaviourally grounded while functionally aligned with CBC.

#### 3.3.3. Early Socialisation and Prevention-Oriented Interventions

The behavioural and attachment theories support early socialisation and prevention-oriented interventions. Behavioural theory explains the emphasis on sensitive periods and early learning, as reflected in programmes such as S.T.A.R. Puppy and the Puppy Foundation. Attachment theory complements this by explaining the role of consistent caregiving, predictability, and emotional security as a “secure base” for dogs [25,56]. This component thus represents a documented convergence of developmental behavioural principles and emotional–relational stability.

#### 3.3.4. Parallel Structures of Standardisation and Customisation

The coexistence of standardised criteria and individualised application lacks an explicit theoretical explanation in official documents. This study interprets this hybrid structure through CBC’s principle of context-based application, which conceptualises behaviour change as adaptive and environment-dependent rather than manual-driven [30,54]. Therefore, CBC provides a coherent interpretive framework for understanding how countries flexibly implement standards across diverse contexts.

#### 3.3.5. Emotion and Relationship-Centred Interaction and Guardian Change

Emotion- and relationship-centred interaction is the domain most directly grounded in HAI and attachment theories. Programmes such as DogKind and RSPCA Australia explicitly emphasise the recognition of emotional signals, stress reduction, and co-regulation [2]. Owners’ emotional regulation and consistency align with attachment theory’s concept of emotional sensitivity, which studies have associated with improved behavioural stability [57,58]. This study applies CBC as a supplementary interpretive lens to explain owners’ cognitive reframing and behavioural execution processes, without positioning it as a formal theoretical foundation.

### 3.4. Summary

Although institutional arrangements vary, dog owner education systems across the five countries consistently draw on behavioural and human–animal interaction theories. Attachment theory provides complementary explanations for emotional and relational mechanisms, while CBC functions as a structured interpretive lens for explaining owner change processes. This layered theoretical integration clarifies how dog owner education operates through multiple, empirically grounded mechanisms rather than through the universal application of a single theory.

## 4. Discussion

This study employed a qualitative multiple-case comparative approach to examine dog owner education programmes in the United States, the United Kingdom, Germany, Japan, and Australia. The findings reveal substantial cross-national variation in institutional and policy structures, alongside consistent commonalities in the psychological and behavioural mechanisms underlying dog owner education. Within-case analyses (RQ1) demonstrated that each country has institutionalised dog owner education through distinct historical, sociocultural, and policy trajectories. In contrast, the cross-case comparison (RQ2) identified five core components that consistently emerged across all cases: owner responsibility and welfare standards, science-based positive reinforcement training, early socialisation and prevention-oriented interventions, a hybrid structure of standardisation and customisation, and emotion- and relationship-centred interaction accompanied by owner change. Notably, these components collectively encompass ethical responsibility, scientific training principles, developmental prevention, structural flexibility, and relational–emotional mechanisms of owner change. Together, these findings suggest that dog owner education systems share psychological and behavioural principles that transcend national policy frameworks.

First, the results indicate that dog owner education systems institutionally embed the emotional and relational mechanisms emphasised in human–animal interaction (HAI) theory and attachment theory. Prior research demonstrates that companion animals reduce stress in humans and support emotional regulation [2,23], and that owner–dog relationships function as sources of reciprocal emotional regulation and bonding [3,24]. Attachment theory further highlights that predictability, emotional sensitivity, and consistency in caregiving promote secure attachment and adaptive behaviour [22,25]. The present study shows that national systems do not treat emotion- and relationship-centred interaction as a byproduct of training; instead, they explicitly translate these principles into educational goals, formal guidelines, and practice-based curricula, reflecting a shift beyond technical skill acquisition towards relational stability and dyadic regulation.

Second, the findings highlight strong alignment between national dog owner education systems and behavioural science. Extensive research has suggested that positive reinforcement-based training is associated with fewer problem behaviours and improved welfare outcomes, while owners’ attitudes and interaction styles play an important role in shaping canine behavioural stability [41,57,59]. Consistent with this evidence, all five countries adopted non-coercive, reinforcement-based approaches, emphasising early socialisation and promoting stress-reduction-oriented interaction. This convergence supports behavioural theory perspectives that conceptualise canine behaviour as emerging from interactions among environmental, emotional, and learning conditions rather than as isolated deficits [32,33].

Third, cross-national comparison clarifies how different governance models operationalise these shared principles. The United States relies primarily on voluntary, private certification; the United Kingdom integrates welfare legislation with private certification; Germany adopts a strongly regulated, mandatory testing system; Japan emphasises municipality-led, community-based education; and Australia operates a hybrid model combining welfare policy and vocational education. Despite these divergent institutional pathways, all five systems organise dog owner education around the same five core components. Notably, assessment and certification mechanisms varied substantially across cases, suggesting that they function as context-specific implementation tools that support, rather than constitute, the core components of dog owner education.

When interpreting cross-national differences in dog owner education systems, it is important to consider the historical context of companion-dog culture in East Asian countries such as Japan and South Korea. In Japan, institutional emphasis on animal welfare and owner responsibility intensified toward the late 1990s, marked by the major revision of the Animal Welfare and Management Act in 1999 and a subsequent long-term decline in dog and cat intake and euthanasia, alongside the gradual professionalisation of nutrition, preventive veterinary care, and everyday management practices [18,60]. Both Japan and South Korea share predominantly small-breed ownership profiles associated with urban living environments, as reflected in kennel club and national survey data [61,62], providing an interpretive background for the relatively compressed emergence of formal dog owner education systems without implying equivalence in educational outcomes or effectiveness [6].

In addition to institutional and policy differences, culturally embedded perspectives on dogs and dog ownership may further shape how dog owner education is received and embodied across national contexts [3,63]. Conceptions of dogs as family members, working animals, or managed companions influence owners’ expectations, caregiving norms, and openness to training philosophies, thereby affecting the relative emphasis placed on emotional, relational, or behavioural components within education programmes [4]. Accordingly, the shared structural principles identified in this study should be understood as operating within culturally specific meaning systems, which may moderate how educational paradigms are interpreted, enacted, and sustained over time.

Fourth, these findings have implications for the ongoing development of dog owner education systems in South Korea. Previous studies documented the absence of a national standard or comprehensive legal framework in Korea, resulting in fragmented provision and wide variability in owners’ knowledge and practices [5,6,64]. Rather than prescribing a direct model transfer, the present study suggests that Korea could benefit from selectively adapting shared principles identified across international cases. In particular, integrating welfare-oriented institutional elements observed in the United Kingdom and Australia with Japan’s community-based, life-contextualised approach may offer a culturally and administratively appropriate direction. Within this context, a coaching-oriented education model grounded in HAI theory and cognitive–behavioural principles may offer a theoretically informed framework for supporting owners’ cognitive, emotional, and behavioural engagement.

Fifth, this study contributes to the reconceptualisation of dog owner education as a psychosocial and coaching-oriented intervention. The analysis did not treat cognitive–behavioural coaching (CBC) as a theory explicitly adopted by national programmes; instead, it employed CBC as an interpretive analytical lens to explain recurring structural features in educational documents, including goal clarification, staged learning processes, feedback mechanisms, and supports for reflection and self-efficacy [31,53,54]. Accordingly, this study does not position CBC as an operational framework for national systems; rather, it uses CBC as a theoretical tool to interpret the mechanisms of owner change embedded within existing educational structures.

Finally, from an academic perspective, this study extends the literature by integrating HAI theory, attachment theory, behavioural science, and coaching psychology within a comparative, system-level framework. Whereas prior research often focused on individual programmes or isolated outcomes, this study conceptualises dog owner education as a multi-layered intervention combining emotional, relational, cognitive, and behavioural dimensions. Methodologically, integrating context-informed multiple-case analysis [35] with pattern-based comparative analysis [50] demonstrates the utility of qualitative comparative research for identifying shared structural principles across national contexts [34,37].

## 5. Practical Implications

This study offers structural and conceptual reference points for considering the future development of dog owner education systems, particularly in contexts such as South Korea, where formalised national frameworks remain limited. Importantly, the following implications are descriptive and exploratory, derived from cross-national comparison of existing systems, rather than claims about educational effectiveness.

First, the cross-case analysis highlights how dog owner education is organised as a tiered and modular system across multiple national contexts. The shared core components identified in this study illustrate that education systems can be structured as multi-layered frameworks rather than single, uniform programmes. From a policy and design perspective, these international cases demonstrate how educational content may be differentiated according to contextual factors such as owners’ caregiving experience, dogs’ age, temperament, socialisation history, and household environments. Modular structures—such as introductory stages focused on responsibility and welfare awareness, intermediate stages addressing socialisation and prevention, and advanced stages emphasising relational interaction—represent one way in which existing systems organise educational pathways. These structures are observed descriptively in programmes such as the United Kingdom’s Good Citizen Dog Training Scheme (GCDTS) and the U.S. Canine Good Citizen (CGC), which combine standardised criteria with context-sensitive application.

Second, the findings draw attention to the institutional presence of emotion- and relationship-centred educational content across countries. The consistent inclusion of emotional awareness, relational understanding, and owner self-reflection within national education frameworks suggests that dog owner education is not limited to technical skill instruction alone. In systems examined in this study, educational materials frequently reference owners’ emotional states, stress responses, and interaction styles as relevant dimensions of dog–human relationships. From a practical standpoint, these observations may inform discussions about expanding the conceptual scope of owner education to include psychosocial dimensions, particularly in contexts where education is predominantly delivered through private or fragmented channels.

Third, the comparative analysis illustrates how early socialisation and prevention-oriented education are supported through institutional mechanisms such as minimum standards, guidelines, and evaluative tools. Across the five countries, governments and professional organisations employ varying combinations of voluntary and mandatory instruments to promote early-stage engagement and continuity of learning. These approaches provide examples of how national systems seek to stabilise educational quality and reduce variability across providers. In the Korean context, such international cases may serve as reference points for considering how baseline guidelines or competency benchmarks could be gradually articulated, without implying direct transferability or uniform application.

Fourth, the study highlights the relevance of process-oriented behaviour change frameworks as analytical lenses for understanding dog owner education. While national systems do not explicitly adopt coaching models, educational structures frequently incorporate staged learning, reflection, feedback, and owner responsibility. In this study, cognitive–behavioural coaching (CBC) was used solely as an interpretive framework to describe these recurring features, not as an evaluative or prescriptive model. As such, CBC-informed concepts may offer a conceptual vocabulary for analysing how owner change is addressed within educational systems, without asserting their effectiveness or necessity.

Finally, the findings underscore the role of broader policy and social support infrastructures in shaping dog owner education. Cross-national comparison shows that owner behaviour is widely recognised as a factor linked to animal welfare and public safety, and that education is often embedded within wider community, municipal, or professional systems. In Korea, international examples may provide descriptive insight into how education could be situated within public services, community programmes, or locally coordinated initiatives, thereby reducing exclusive reliance on private provision.

Overall, the practical implications of this study are best understood as structural insights rather than outcome-based recommendations. By comparing how dog owner education is organised across national systems, this study reframes owner education as a socially embedded, multi-level educational structure. These observations may serve as a conceptual foundation for future policy discussions, programme development, and empirical research while remaining distinct from claims about educational effectiveness or causal impact.

## 6. Limitations and Directions for Future Research

Despite its contributions, this study has several limitations that warrant consideration.

First, the comparative analysis focused on countries with relatively well-established dog owner education systems. This focus limits the generalisability of the findings to contexts where education frameworks remain emergent or weakly institutionalised.

Second, the study relied primarily on documentary data, including policy documents, institutional guidelines, and programme manuals. Consequently, the analysis did not directly examine the lived experiences or perspectives of dog owners, trainers, and educators. Future research could address this limitation by incorporating interviews, observations, or survey-based methods to capture how education systems function in practice.

Third, the study emphasised institutional structures and theoretical characteristics rather than the empirical effectiveness of individual programmes. Accordingly, this study does not provide direct evidence of behavioural or welfare outcomes associated with specific interventions.

Fourth, because the national education system served as the unit of analysis, the study could not fully account for how dog-specific variables—such as breed, temperament, age, or prior socialisation—shape educational outcomes.

Despite these limitations, this study makes a meaningful contribution by offering a comparative framework that integrates institutional, psychological, and behavioural perspectives. By identifying shared core components across countries, the study reconceptualises dog owner education as a multi-layered educational system rather than as a collection of isolated programmes and establishes a conceptual foundation for developing a context-appropriate Korean model.

Future research should build on this framework through implementation studies, longitudinal designs, and mixed-methods approaches to examine whether and how dog owner education and coaching-based interventions are associated with sustained changes in owner behaviour, dog welfare, and human–animal relationships over time.

## 7. Conclusions

This study offers a comparative, system-level understanding of dog owner education by examining how such education is institutionally organised and conceptually framed across five national contexts. Rather than treating owner education as a collection of isolated training programmes, the findings position it as a multi-layered educational system that integrates ethical responsibility, behavioural science, developmental prevention, and relational–emotional engagement within broader policy and social infrastructures.

Across diverse governance models and cultural settings, dog owner education consistently operates through mechanisms that address not only technical skills but also owners’ cognitive, emotional, and relational orientations toward their dogs. By drawing on human–animal interaction (HAI) theory, attachment theory, and behavioural science, this study highlights that owner sensitivity, consistency, and relational engagement are institutionally recognised as important for canine welfare and behavioural stability. These insights underscore the importance of understanding owner education as a psychosocial intervention embedded within human–animal relationships, rather than as a purely instructional or compliance-oriented activity.

Conceptually, the study demonstrates the analytical value of cognitive–behavioural coaching (CBC) as an interpretive lens for examining how owner change is implicitly structured within existing education systems. While CBC is not proposed as an operational model for national programmes, its emphasis on staged learning, reflection, feedback, and self-regulation provides a useful framework for interpreting how educational systems support sustained owner engagement and behavioural adaptation.

For South Korea, the comparative findings offer structural reference points rather than prescriptive solutions. In a context where dog owner education remains fragmented and largely dependent on private provision, the study suggests that future system development may benefit from selective adaptation of internationally shared principles, informed by local cultural meanings, social conditions, and administrative capacities. Importantly, these insights do not imply equivalence of outcomes across countries but instead support context-sensitive interpretation and policy reflection.

Overall, this research advances the field by reframing dog owner education as a system-level, psychosocial, and relationally grounded educational structure. Methodologically, it illustrates the contribution of qualitative multiple-case comparative analysis to identifying shared educational principles across national contexts. Substantively, it establishes a conceptual foundation for future empirical work—particularly implementation studies, longitudinal designs, and mixed-methods research—aimed at examining whether and how dog owner education is associated with sustained changes in owner behaviour, canine welfare, and human–animal relationships over time.

## Figures and Tables

**Table 1:** Composition of analytical data sources for dog owner education by country.

Country	Official/Legal Documents (Primary)	Policy/Institutional Materials (Primary)	Academic Studies (Secondary/Interpretive)	Total
United States	AKC S.T.A.R. Puppy Programme; AKC Canine Good Citizen (CGC)	APDT Training Guidelines; Animal Behaviour College Curriculum Overview	Johnson & Wynne (2024) [41]	5
United Kingdom	Animal Welfare Act 2006; Five Welfare Needs Framework	The Kennel Club Good Citizen Dog Training Scheme (GCDTS); RSPCA DogKind Programme	Philpotts et al. (2019, 2024a, 2024b) [13,14,43]	6
Germany	Tierschutz-Hundeverordnung (BMEL, 2021) [15]; NHundG Sachkundenachweis (Niedersachsen)	VDH Hundefuhrerschein Guidelines	Küeper & Merle (2021) [16]; Rauch et al. (2021) [17]	5
Japan	Ministry of the Environment Companion Animal Guidelines	Tokyo Animal Welfare Centre Owner Education Materials; JAWS Educational Resources	Yamada et al. (2019) [19]	4
Australia	Australian Animal Welfare Strategy Framework	RSPCA Australia Education Resources; Delta Institute Cert IV Curriculum	Napier et al. (2021) [21]	4

**Table 2:** Institutional characteristics of dog owner education programmes by country (RQ1).

Analytical Category	United States (US)	United Kingdom (UK)	Germany (DE)	Japan (JP)	Australia (AU)
Institutional structure	Private-led self-regulatory model: Limited federal mandates; private standards (e.g., AKC, APDT) substantially shape educational direction	Law–private standard linkage model: Based on the Animal Welfare Act 2006 and Five Welfare Needs; The Kennel Club and RSPCA disseminate institutional norms	Statutory regulation-centred model: Based on Tierschutzgesetz and subsidiary regulations; qualification and regulatory intensity institutionalised at the state (Länder) level	Decentralised local-government-led model: Municipality-designed and operated programmes grounded in Ministry of the Environment guidelines	Public–professional integrated model: National/state policy frameworks combined with vocational education (VET) and welfare organisations (e.g., RSPCA)
Educational objectives and staged structure	Tiered training pathway (S.T.A.R. Puppy → CGC → advanced levels): Socialisation, basic manners, public conduct norms, and responsible ownership	Welfare-, emotional-, and social responsibility-integrated multi-stage structure (Puppy Foundation → Bronze → Silver → Gold) including relational components	Statutory responsibility-focused sequence: Knowledge (management, law, behaviour) followed by practical skills (control, public behaviour) via education–assessment–application	Daily-life adaptation and prevention-oriented modular structure: Short units such as early socialisation classes, adult manners, and problem behaviour consultations	Science-based and welfare-centred integrated objectives: Socialisation, environmental management, early prevention, stress-signal interpretation; partially linked to professional training pathways
Delivery system and implementing actors	Private professionals as primary providers: AKC-certified trainers, APDT professionals, and training institutions (e.g., ABC)	Public–private linkage: Central guidance and accreditation with local implementation by accredited educators; RSPCA campaigns and education operate in parallel	Public oversight with certified trainers: State governments or authorised bodies supervise; separation of educators and evaluators (varies by state)	Local government and community collaboration: Public officials, veterinarians, and private trainers cooperate; relatively high emphasis on consultation and individualised guidance	Integration of public agencies, welfare organisations, and vocational education: RSPCA materials and education combined with trainer training via TAFE/Delta; state-level quality assurance mechanisms
Assessment and certification	Voluntary participation with quasi-standardisation: CGC functions as a nationwide benchmark; ethical and competency standards maintained by professional bodies	Clearly defined stage-based achievement criteria: GCDTS progression; some programmes assess emotional and relational components	Conditionally mandatory assessments (statutory): Sachkundenachweis for owners meeting specific criteria (theory + practice), administered by certified evaluators/institutions	Limited national standardisation: Practical evaluations such as course completion, counselling outcomes, and follow-up monitoring at the municipal level	Multi-layered assessment structure: National qualifications (VET) for professionals alongside competency-based and informal achievement verification in owner education
Psychological foundations	Positive reinforcement and learning theory emphasis, with relational quality, emotional understanding, and owner emotion regulation framed as mediators of behaviour change	Welfare- and fear-free orientation: Explicit focus on signal interpretation, emotional regulation, and stress reduction as pathways to behaviour change	Strong emphasis on control and safety; increasing recognition of predictability, consistency, and emotional stability as relational factors	Life-oriented psychological approach: Emphasis on emotional signals, stress reduction, and prevention, with practice prioritised over explicit theoretical framing	Integrated behavioural science and emotional–relational approach: Stress signals and environmental management emphasised; learning theory linked to instructional competence
Standardisation–individualisation balance	National standard modules with high contextual customisation (living environment, ownership style, problem type)	Strict standardised stages coexisting with welfare-based individualisation (adjustments for emotion, stress, and environment)	Strong statutory standardisation with limited customisation (standards prioritised; some adjustment by temperament or risk level)	Weak national standardisation with high life-context customisation (municipality- and case-centred adjustments)	Common guidelines with region- and target-specific implementation (standard modules plus practice and feedback to reflect individual differences)
Publicness, ethics, and responsibility	Social-norm-based responsibility enhancement: Ethical standards and prohibition of coercion via private self-regulation; CGC incorporates responsible behaviour	Strong institutionalisation of legal duties and welfare norms: Duty of care and Five Needs structurally embedded in education and assessment	Strongest statutory responsibility and public safety norms: Responsibility institutionalised through qualification and regulation	Community coexistence-oriented publicness: Emphasis on safety, coexistence, and prevention (lower statutory intensity)	Welfare principles and responsible ownership emphasised: Welfare requirements (including emotional welfare) are defined as the owner’s responsibility; ethical standards are embedded in professional education

Note. AKC = Association of Professional Dog Trainers, APDT = American Kennel Club.

**Table 3:** Documentary evidence matrix of shared core components across countries (RQ2).

Shared Core Components	United States	United Kingdom	Germany	Japan	Australia
Guardian Responsibility and Welfare Standards	AKC Canine Good Citizen (CGC); APDT Guidelines	Animal Welfare Act 2006; GCDTS; RSPCA DogKind	Tierschutz-Hundeverordnung; NHundG	Ministry of the Environment (MoE) Guidelines; Tokyo Animal Welfare Centre	Australian Animal Welfare Strategy (AAWS) Framework; RSPCA Australia
Science-Based Training Grounded in Positive Reinforcement	AKC Programmes; APDT Standards; Animal Behaviour College (ABC)	GCDTS; DogKind	VDH Training Guidelines	JAWS Educational Materials	Delta Institute Certificate IV; RSPCA Australia
Early Socialisation and Prevention-Oriented Interventions	S.T.A.R. Puppy Programme	Puppy Foundation; GCDTS	Sachkundenachweis Preparation Programmes	Local-Government-Led Programmes	Early Socialisation Guides
Parallel Structures of Standardisation and Customisation	National AKC Modules with Local Delivery	National Standards with Welfare-Based Adaptation	Legal Standards with Limited Flexibility	Localised Guidance and Case-Based Adjustment	National Framework with Regional Adaptation
Emotion and Relationship-Centred Interaction and Guardian Change	CGC; Owner Attitude and Responsibility Components	DogKind; Welfare-Oriented Education	Emerging Emphasis in Educational Discourse	Daily Interaction and Caregiving Guidance	RSPCA Programmes Emphasising Emotional Literacy

Note. AKC = American Kennel Club, CGC = Canine Good Citizen.

**Table 4:** Theory-based application structure for shared core components (RQ3).

Shared Core Components	Theories Directly Reflected in Documentary Evidence	Theories Applied as Interpretive Analytical Lenses
Guardian Responsibility and Welfare Standards	Human–Animal Interaction (HAI) Theory	Cognitive–Behavioural Coaching (CBC)
Science-Based Training Grounded in Positive Reinforcement	Behavioural Theory	Cognitive–Behavioural Coaching (CBC)
Early Socialisation and Prevention-Oriented Interventions	Behavioural Theory; Attachment Theory	—
Parallel Structures of Standardisation and Customisation	—	Cognitive–Behavioural Coaching (CBC)
Emotion and Relationship-Centred Interaction and Guardian Change	Human–Animal Interaction (HAI) Theory; Attachment Theory	Cognitive–Behavioural Coaching (CBC)

## Data Availability

This study is based on the analysis of publicly available documents and published literature. No new datasets were generated or analysed. Data sharing is not applicable to this article.

## Appendix A

**Table A1:** National document corpus for dog owner education.

Country	Document Title	Issuing Body/Author	Year	Document Type	Analysis Stage	Rationale (Representativeness/Authority)
United States	S.T.A.R. Puppy Programme Guide	American Kennel Club (AKC) [11]	n.d.	Institutional programme manual	Primary	De facto national standard for early-stage dog owner education in the absence of federal mandates
United States	Canine Good Citizen (CGC) Programme	American Kennel Club (AKC) [10]	n.d.	Certification and assessment standard	Primary	Representative certification evaluating owner responsibility and public safety competencies
United States	Training Guidelines	Association of Professional Dog Trainers (APDT) [39]	n.d.	Professional guidelines	Primary	Authoritative expert standards emphasising positive reinforcement-based training
United States	Curriculum Overview	Animal Behaviour College (ABC) [40]	n.d.	Educational curriculum	Primary	Reflects standardised delivery structures widely used in private owner and trainer education
United States	Dog Owner Education and Behaviour Outcomes	Johnson & Wynne [41]	2024	Peer-reviewed article	Secondary	Used to interpret behavioural and welfare-related effects of owner education
United Kingdom	Animal Welfare Act	UK Government [52]	2006 (in force)	Legislation	Primary	The highest-level legal framework defining the owner’s statutory duty of care
United Kingdom	Five Welfare Needs Framework	DEFRA/RSPCA [48]	n.d.	Policy framework	Primary	Core welfare standards underpinning owner education and animal welfare practice
United Kingdom	Good Citizen Dog Training Scheme (GCDTS)	The Kennel Club [12]	n.d.	Institutional programme	Primary	Representative national, stage-based standard for dog owner education
United Kingdom	DogKind Programme	RSPCA [43]	n.d.	Institutional guidelines	Primary	Welfare-, emotion-, and relationship-centred owner education model
United Kingdom	Owner Education and Welfare Outcomes	Philpotts et al. [13,14]	2019, 2024	Peer-reviewed articles	Secondary	Empirical interpretation of links between owner education, welfare awareness, and behaviour change
Germany	Tierschutzgesetz (German Animal Welfare Act)	BMEL [15]	2021	Federal Law	Primary	Comprehensively defines owners’ duty of care and fundamental principles of animal welfare.
Germany	Tierschutz-Hundeverordnung (German Animal Welfare Dog Ordinance)	BMEL [15]	2021	Regulation	Primary	Federal-level legal regulation defining owner responsibility and welfare standards
Germany	Niedersächsisches Hundegesetz (NHundG; Lower Saxony Dog Ownership Act)	State of Lower Saxony [51]	2011 (in force)	State law	Primary	Most clearly institutionalised example of mandatory owner competency certification (Sachkundenachweis)
Germany	Hundeführerschein Guidelines (dog owner competency certification guidelines)	Verband für das Deutsche Hundewesen (VDH) [15]	n.d.	Private certification guidelines	Primary	De facto national private standard complementing statutory regulation
Germany	Public Acceptance of Owner Regulation	Küeper & Merle [16]	2021	Peer-reviewed article	Secondary	Interprets social acceptance of regulation-based owner education.
Germany	Behavioural Impact of Dog Ownership Regulation	Rauch et al. [17]	2021	Peer-reviewed article	Secondary	Analysis of behavioural effects of regulatory owner education policies
Japan	Companion Animal Welfare Guidelines	Ministry of the Environment [18]	n.d.	Government guidelines	Primary	Central government guidance outlining owner responsibility and welfare principles
Japan	Responsible Ownership Guidelines	Tokyo Animal Welfare Centre [18]	n.d.	Municipal guidelines	Primary	Representative example of community-based, daily-life-oriented owner education
Japan	Educational Resources	Japan Animal Welfare Society (JAWS) [44]	n.d.	NGO materials	Primary	Reflects public–private collaboration in owner education delivery
Japan	Determinants of Owner Behaviour Choice	Yamada et al. [19]	2019	Peer-reviewed article	Secondary	Empirical study on psychological and contextual factors influencing owner behaviour
Australia	Australian Animal Welfare Strategy (AAWS)	Australian Government [20]	n.d.	National policy framework	Primary	National-level framework articulating animal welfare and owner responsibility principles
Australia	Ownership & Education Resources	RSPCA Australia [42]	n.d.	Institutional guidelines	Primary	Widely used public-sector owner education standards
Australia	Certificate IV in Animal Behaviour and Training	Delta Institute [47]	n.d.	Vocational education curriculum	Primary	Standardised, evidence-based training structure for owners and professionals
Australia	Delivery Modes of Owner Education	Napier et al. [21]	2021	Peer-reviewed article	Secondary	Interpretation of delivery modes, including online owner education

## Appendix B

**Table A2:** Summary of document screening procedures.

Stage	Screening Procedure	Number of Documents (n)	Notes
Stage 1	Initial search results (databases + institutional websites)	120	Scopus, RISS, Google Scholar, and official government and institutional websites
Stage 2	Removal of duplicate records	30 removed	Duplicate publications of the same document, including revised or updated versions
Stage 3	Title and abstract screening	90 → 60	Relevance to research objectives and direct relevance to dog owner education
Stage 4	Source credibility assessment	60 → 40	Exclusion of non-official materials, commercial promotional content, and personal media
Stage 5	Publication year criterion applied	40 → 32	Documents published prior to 2015 were generally excluded
Stage 6	Application of exception criteria	Maintained	Legal documents and national strategies or policy frameworks with structural influence on institutional formation were retained regardless of publication year
Stage 7	Full-text review	32 → 24	Comparative relevance and national representativeness assessed
Final	Final document set included in analysis	24	8 legal and regulatory documents; 8 policy and institutional documents; 8 academic studies

## Appendix C

**Table A3:** Codebook: operational definitions and document-based examples of analytical categories.

Analytical Category	Operational Definition	Coding Criteria	Document-Based Examples (Extracted or Summarised)	Source Documents
1. Institutional Structure	The extent to which dog owner education is formally institutionalised through legislation, administrative regulations, public policy, or authoritative standards issued by recognised public or private institutions, focusing on its legal and administrative foundations	Explicit mention of owner responsibility in legal or policy documents Existence of national or institutionally authoritative standards Formal linkage between education systems and legal or regulatory frameworks	Owner duties of care and welfare responsibilities are formally defined in law or official guidelines and linked to education and certification systems	Animal Welfare Act 2006; NHundG (Lower Saxony); AKC Canine Good Citizen (CGC)
2. Educational Goals and Programme Structure	The stated goals of dog owner education and the presence of structured programme designs, including staged or sequential learning components (e.g., foundational, intermediate, and advanced levels)	Explicit articulation of educational objectives Presence of structured or staged programmes Emphasis on early-stage owner intervention	Dog owner education programmes are structured around responsibility awareness, basic behaviour guidance, socialisation, and relationship formation	Kennel Club Good Citizen Dog Training Scheme (GCDTS); AKC S.T.A.R. Puppy
3. Delivery Systems and Implementing Bodies	The organisational arrangements through which dog owner education is delivered, including implementing bodies (public vs. private) and modes of delivery (e.g., in-person, online)	Identification of public and/or private implementing bodies Differentiation of delivery modes Criteria for educator qualifications or professional competence	Dog owner education is delivered through a division of roles between public authorities and private professional organisations	RSPCA Australia; Tokyo Animal Welfare Centre
4. Assessment and Certification Systems	The presence and structure of formal mechanisms used to assess and certify owner competence, including examinations, evaluations, or certification systems	Existence of formal assessment or testing procedures Operation of certification or accreditation systems Degree of mandatory versus voluntary participation	Owner competence is assessed through formal evaluations, with some jurisdictions mandating certification	Sachkundenachweis (Germany); AKC CGC Evaluation
5. Psychological Foundations	The mechanisms underlying emotional, cognitive, and behavioural change reflected in dog owner education, including how owners’ emotional regulation, perceptions, and behavioural choices are linked to canine behaviour stability and relationship quality	References to emotional understanding or regulation Emphasis on changes in owner perceptions or attitudes Articulation of psychological principles underlying behaviour change	Owners’ emotional regulation and attitudinal change are framed as contributing to behavioural stability and welfare outcomes in dogs.	RSPCA DogKind Programme; Ministry of the Environment (Japan) Guidelines
6. Standardisation–Customisation Balance	The coexistence of standardised national or institutional criteria with flexible application adapted to owners’ characteristics, dogs’ temperaments, living environments, and broader sociocultural contexts	Presence of standardised core criteria Allowance for individualised adaptation Consideration of environmental, temperamental, or contextual factors	Shared educational standards are maintained while the application is adjusted according to the owner’s and the dog’s characteristics	GCDTS Framework; RSPCA Australia
7. Ethical Responsibility and Welfare Orientation	The extent to which dog owner education frames ownership as an issue of ethical responsibility, animal welfare, and social accountability, rather than solely an individual choice	Explicit references to animal welfare and ethical caregiving Emphasis on owners’ moral and social responsibility Linkage between ownership, public safety, and social coexistence	Owner responsibility is presented as directly linked to animal welfare and community safety, reflecting an ethical orientation towards guardianship.	Animal Welfare Act 2006; German Municipal Regulations
